# KEMFF: A Knowledge-Enhanced and Multidimensional Feature Fusion Model for Aspect-Based Sentiment Analysis

**DOI:** 10.3390/e28080930

**Published:** 2026-08-19

**Authors:** Shuangshuang Yang, Peilun Liu, Wenlong Zhu

**Affiliations:** 1 College of Teacher Education, Qiqihar University, Qiqihar 161006, China; 2 College of Computer and Control Engineering, Qiqihar University, Qiqihar 161006, China; 3 Heilongjiang Key Laboratory of Big Data Network Security Detection and Analysis, Qiqihar University, Qiqihar 161006, China

**Keywords:** sentiment analysis, pre-trained model, graph convolutional network, knowledge enhancement, feature fusion

## Abstract

Aspect-based Sentiment Analysis (ABSA) is a fine-grained sentiment classification task that aims to predict the sentiment polarity associated with aspect terms in sentences. Traditional methods based on syntactic and semantic dependency trees are insufficient for capturing contextual sentence features. To address this, we propose a Knowledge-Enhanced and Multidimensional Feature Fusion (KEMFF) model for ABSA, which captures sentiment feature representations across multiple dimensions, including syntax, semantics, and knowledge. First, the pre-trained model RoBERTa is used to obtain embeddings of sentences and aspect terms. Then, a syntactic dependency parser and a graph convolutional network are utilized to learn syntactic features. Meanwhile, an Abstract Meaning Representation (AMR)-based parser is employed to construct semantic relations, and axial attention is used to aggregate incoming and outgoing semantic dependencies. Furthermore, external knowledge is embedded, and an attention mechanism is employed to obtain aspect-specific knowledge representations, thereby complementing syntactic and semantic representations with external lexical knowledge. Finally, multidimensional features are fused and passed to a softmax classifier for predicting sentiment polarities. Unlike previous models that mainly focus on either syntax–semantic fusion or knowledge-enhanced graph propagation, KEMFF explicitly models syntax, semantics, and lexical knowledge in three parallel branches and aligns them into a unified aspect-level representation. Experiments on Laptop14, Restaurant14, and Twitter datasets show that KEMFF achieves the best performance among the compared baselines on Laptop14 and Restaurant14, and it obtains competitive results on Twitter.

## 1. Introduction

**Figure 1:**
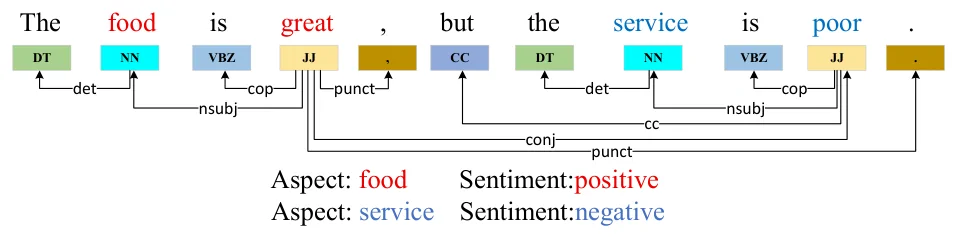
Example sentence with a dependency tree, where positive terms are colored in red and negative terms are colored in blue.

Aspect-based sentiment analysis (ABSA) is a well-established fine-grained sentiment analysis task that has attracted significant attention in the natural language processing community [1]. Unlike sentence-level sentiment classification, which assigns a holistic polarity to an entire sentence, ABSA aims to identify the polarity of the sentiment (positive, neutral, or negative) expressed towards specific aspect terms appearing in the text [2]. For example, as shown in Figure 1, in the sentence “The food is great, but the service is poor.”, the model should correctly associate the positive sentiment with the aspect “food” and the negative sentiment with “service”. This fine-grained capability makes ABSA crucial for extracting actionable insights from user-generated content, such as product reviews and social media posts.

Over the years, ABSA methods have evolved significantly, primarily driven by advances in deep neural networks. According to the type of feature representation learned, these methods can be broadly categorized into semantic-based and syntax-based methods. Early deep learning approaches leveraged Long Short-Term Memory (LSTM) networks alongside attention mechanisms to capture aspect-specific contextual semantics [3,4]. However, these semantic-based models often struggle when sentences contain multiple aspects with conflicting polarities, as the attention mechanism may erroneously focus on irrelevant opinion words. To overcome this limitation, syntax-based methods have turned to Graph Neural Networks (GNNs). By constructing graphs over dependency trees, models such as ASGCN [5] and DualGCN [6] utilize Graph Convolutional Networks (GCNs) to explicitly model syntactic relationships between aspect terms and their corresponding opinion words. While effective in establishing syntactic connections, these approaches often rely on rigid tree structures that may contain noisy or ambiguous edges.

To further enhance the contextual understanding of ABSA models, researchers have explored two additional directions: external knowledge integration and large-scale pre-training. Knowledge-enhanced ABSA methods, such as SK-GCN [7] and K-BERT [8], incorporate commonsense and sentiment lexicons to provide supplementary relational cues that are not explicitly present in the text, thereby alleviating issues related to implicit sentiment expression. More recently, the advent of Pre-trained Language Models (PLMs), such as BERT [9] and RoBERTa [10], has revolutionized the field by providing rich contextual embeddings that significantly boost performance across all ABSA subtasks. Despite these advances, a common challenge persists: models tend to prioritize one type of information (e.g., semantics, syntax, or knowledge) while failing to holistically integrate these heterogeneous signals in an effective manner.

To address the limitations of isolated feature learning, we propose a Knowledge-Enhanced and Multidimensional Feature Fusion (KEMFF) model for ABSA. KEMFF learns complementary textual representations in parallel along three dimensions: syntax, semantics, and lexical knowledge. In the syntactic dimension, a dependency parser and a two-layer GCN are employed to analyze grammatical structures and compute syntactic dependency relations. In the semantic dimension, an abstract semantic parser constructs a token-pair-level semantic relation tensor, and axial attention aggregates outgoing and incoming semantic relations to capture abstract semantic dependencies beyond surface syntax. Crucially, in the knowledge dimension, external linguistic resources are incorporated through an attention mechanism to produce aspect-specific knowledge features. By unifying these parallel streams through a dedicated feature fusion module, KEMFF constructs a robust and multi-view characterization of aspect-sentiment relationships.

Compared with existing models, the novelty of KEMFF lies not in simply stacking previously proposed components, but in the following integrated design: it explicitly separates syntax, semantics, and lexical knowledge into three parallel representation spaces; it introduces an Abstract Meaning Representation (AMR)-based semantic relation tensor with axial attention to distinguish incoming and outgoing semantic dependencies; it incorporates WordNet knowledge through aspect-sentence attention rather than explicit knowledge graph propagation; and it aligns heterogeneous token-level and aspect-level representations into a unified aspect-level vector for sentiment classification. In summary, the main contributions of this paper are as follows:

- We propose KEMFF, a knowledge-enhanced, multidimensional feature fusion framework for ABSA. The model jointly exploits contextual, syntactic, semantic, and external knowledge representations to improve aspect-specific sentiment classification.
- We introduce a parallel syntax–semantics–knowledge modeling architecture. The syntactic branch captures explicit grammatical dependencies through a GCN, the semantic branch models AMR-based abstract semantic relations using axial attention and semantic-aware attention, and the knowledge branch incorporates WordNet embeddings through aspect-aware attention.
- We design a unified feature fusion module to align and combine heterogeneous representations from syntactic, semantic, and knowledge dimensions, enabling the model to characterize aspect–opinion relations from multiple complementary views.
- Experimental results on Laptop14, Restaurant14, and Twitter benchmark datasets demonstrate that KEMFF achieves the best performance among the compared baselines on Laptop14 and Restaurant14, and it obtains competitive results on Twitter, validating the effectiveness of the proposed model.

The remainder of this paper is organized as follows: Section 2 reviews the related work on ABSA, categorizing existing methods into GNN-based and knowledge-enhanced approaches. Section 3 details the architecture and methodology of the proposed KEMFF model. Section 4 presents the experimental results, including comparative analysis, ablation studies, and case studies. Finally, Section 5 concludes the paper and discusses potential directions for future work.

## 2. Related Work

In this section, we provide a comprehensive overview of related work in ABSA, categorized into two major research directions: GNN-based approaches and knowledge-enhanced methods. This taxonomy reflects the evolution from traditional deep learning models to advanced architectures that incorporate graph structures and external knowledge. In addition to reviewing representative studies, we further clarify the differences between the proposed KEMFF and closely related ABSA models.

### 2.1. GNN-Based ABSA

GNNs, particularly Graph Convolutional Networks (GCNs), have become a cornerstone of modern aspect-based sentiment analysis owing to their capacity for explicit syntactic and semantic dependency modeling. These architectures typically transform dependency parse trees into graph structures, enabling the propagation of information between aspect terms and opinion words across long distances [11,12].

A foundational contribution in this area is ASGCN [5], which applies multi-layer GCNs over dependency trees to aggregate neighborhood features and encode syntactic regularities for aspect-specific classification. Extending this line of work, DualGCN [6] introduced a dual-channel design that separately processes syntactic and semantic graphs, illustrating that fusing complementary structural signals can substantially improve model performance. Unlike DualGCN, which focuses mainly on syntactic and semantic graph modeling, KEMFF further introduces a knowledge enhancement module based on WordNet. To alleviate the influence of noisy or sentiment-irrelevant dependency edges, Zhao et al. [13] devised RDGCN, a reinforced dependency GCN that leverages a policy network to assign explicit importance scores to syntactic connections, thereby enabling selective weighting of dependency edges based on their sentiment relevance.

Several works have extended GNN-based methods to incorporate richer linguistic features. Chen et al. [14] developed EMC-GCN, a multi-channel graph convolutional network that integrates part-of-speech relationships, dependency tree distances, and relative position distances through multiple parallel channels. Similarly, Mei et al. [15] proposed DGLM, which combines disentangled linguistic representation learning, supervised disentangling, and a cross-linguistic routing mechanism to enhance aspect-level representations.

Beyond dependency-based GNNs, recent studies have explored more flexible graph and attention-based architectures for ABSA. For example, TextGT [12] introduces graph transformer structures to strengthen global relational modeling, while DMAN [16] focuses on dynamic multi-granularity feature attribution for aspect-aware sentiment prediction. These models improve the interaction between contextual words and aspect terms from different perspectives. In contrast, KEMFF does not mainly aim to design a new graph transformer or dynamic attribution mechanism. Instead, it explicitly separates syntax, semantics, and external lexical knowledge into three parallel representation views and fuses them after view-specific representation learning.

AMR has also been introduced into ABSA to capture high-level semantic relations that are difficult to represent using surface dependency trees. APARN [17], for instance, focuses on aggregating AMR paths to enhance aspect-level sentiment representation. Different from APARN, KEMFF does not rely solely on AMR path aggregation. It represents AMR information as token-pair-level semantic relations and adopts axial attention to separately aggregate outgoing and incoming AMR-based semantic dependencies. This design enables the model to distinguish between different semantic directions in the AMR graph.

For complex compound ABSA tasks, graph-based methods have been adapted to model multiple sentiment elements jointly. The SpanGCN model [18] leverages dependency-enhanced graph convolution to refine span-level representations for aspect sentiment triplet extraction. Additionally, heterogeneous graph neural networks have been explored to capture diverse relationships among aspects, opinions, and sentiment polarities within a unified graph structure [19].

Despite these successes, GNN-based ABSA models rely heavily on syntactic dependency parsers to construct the underlying graph topology. However, dependency parsers are prone to inaccuracies, especially when processing informal and noisy contexts in social media and online reviews [20].

In summary, most GNN-based ABSA models depend strongly on dependency parsers. Parser errors may propagate into downstream sentiment classification, especially in noisy social media texts. Moreover, syntactic dependency trees mainly capture grammatical relations and may not fully represent abstract semantic dependencies. This motivates us to introduce AMR-based semantic structures as a complementary semantic view.

### 2.2. Knowledge-Enhanced ABSA

External knowledge bases provide valuable commonsense and linguistic information that can compensate for the limited contextual understanding of purely data-driven models. Knowledge-enhanced ABSA methods integrate resources such as SenticNet [21], WordNet [22], and SentiWordNet [23] to enrich semantic representations and improve sentiment inference.

A seminal contribution in this area is KGAN [24], which applies multi-view representation learning over context, syntax, and commonsense knowledge from WordNet, employing a hierarchical knowledge fusion mechanism to selectively integrate external signals for aspect-specific classification. Extending this paradigm, KA-GCN [25] introduced a knowledge-enabled RoBERTa architecture that aligns heterogeneous embeddings from SentiWordNet with transformer token representations through syntactic graph expansion, demonstrating that bridging the gap between PLM tokenization and knowledge graph structures substantially improves sentiment awareness. Compared with KGAN and KA-GCN, which rely more heavily on knowledge graph construction or knowledge-enhanced graph propagation, KEMFF does not perform explicit knowledge subgraph propagation. Instead, WordNet embeddings are concatenated with RoBERTa representations to form knowledge-enhanced aspect and sentence features, which are then used to compute aspect–sentence attention for obtaining aspect-specific contextual representations.

To mitigate the issue of inefficient knowledge retrieval and noisy knowledge injection, Liu et al. [26] devised DGGCN, a dual-gated graph convolutional network that leverages contextual affective knowledge from SenticNet to selectively filter sentiment-relevant information through gated propagation mechanisms, thereby enabling adaptive weighting of external knowledge based on its semantic relevance. Similarly, DSSK-GAN [27] proposed a bifold-GCN architecture that dynamically constructs sentiment-specific nodes alongside static knowledge graphs, illustrating that the joint modeling of dynamic contextual sentiment and static relational knowledge enhances robustness against parsing errors. DAGF [28] proposes a simple form of binary polarity labels from a lexicon to augment syntactic edges and enhance semantic representations. Compared with these models, KEMFF treats lexical knowledge as an independent representation view parallel to syntax and semantics. The knowledge representation is learned and fused after view-specific modeling, rather than being directly embedded into the syntactic graph propagation process.

Several works have extended knowledge-enhanced methods to incorporate richer psychological and multi-granularity features. For example, VADGAT [29] explored triple-dimensional affective knowledge from psychology (Valence-Arousal-Dominance) through graph attention networks to capture fine-grained emotional states. Zhong et al. [30] constructed a four-level multi-granularity extraction module to alleviate the underutilization of fine-grained features in ABSA and introduced implicit psychological sentiment prior knowledge.

To better position KEMFF relative to previous studies, Table 1 compares representative ABSA models from the perspectives of PLM, syntactic modeling, semantic modeling, external knowledge, and fusion strategy.

As shown in Table 1, existing ABSA models have explored syntactic modeling, semantic modeling, knowledge enhancement, and multi-view representation learning from different perspectives. However, most of them emphasize one or two information sources. In contrast, KEMFF explicitly decomposes ABSA representation learning into three complementary dimensions: syntax, semantics, and lexical knowledge. These heterogeneous representations are modeled in parallel and then aligned into a unified aspect-level representation for sentiment prediction.

## 3. Methods

**Figure 2:**
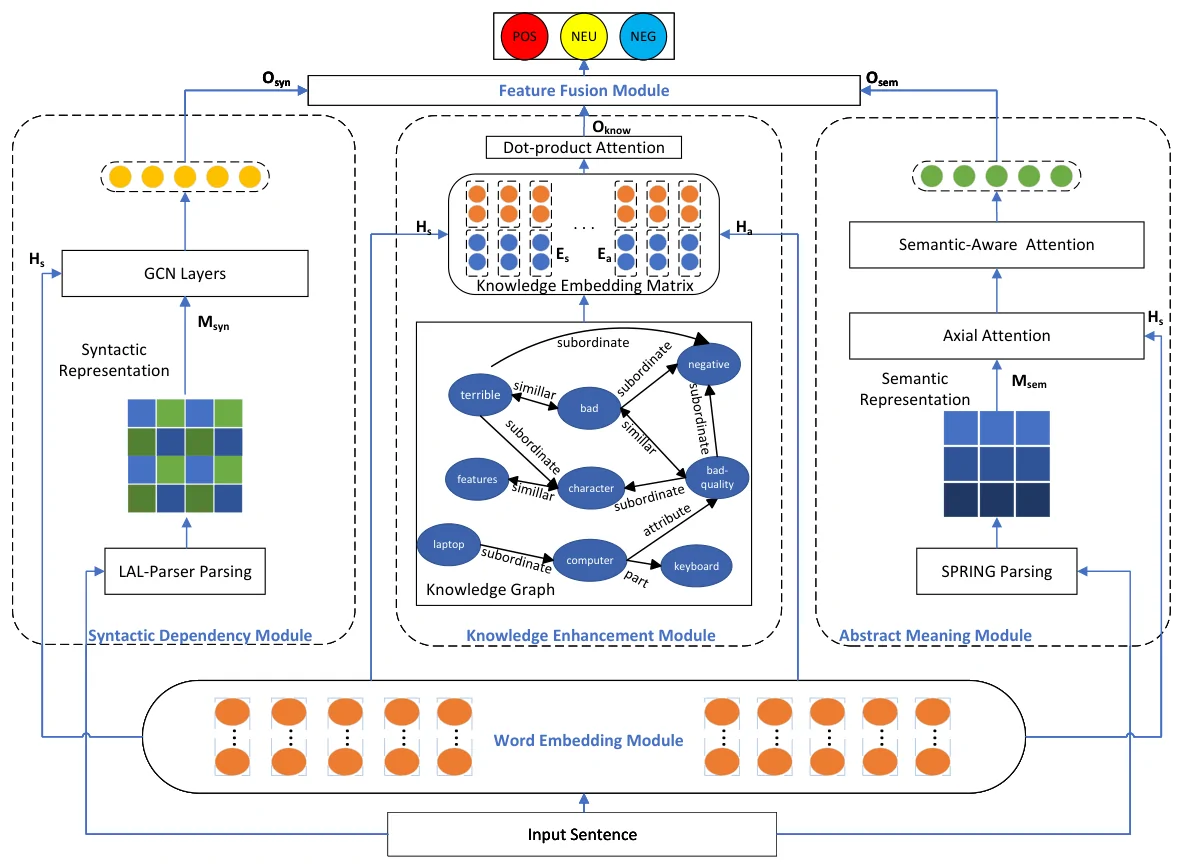
The framework of the KEMFF model.

In the ABSA task, the input consists of a sentence–aspect pair denoted as (S,A), where $S=\{w_{1},w_{2},\ldots ,w_{\tilde{n}}\}$ denotes the sentence and $A=\{a_{1},a_{2},\ldots ,a_{\tilde{m}}\}$ denotes the aspect term with *A* being a subsequence of *S*. The objective is to determine the sentiment polarity (positive, neutral, or negative) toward each aspect term. To address this, we propose the Knowledge-Enhanced and Multidimensional Feature Fusion (KEMFF) model, whose architecture is illustrated in Figure 2. The framework consists of five main modules: word embedding, syntactic dependency, abstract meaning, knowledge enhancement, and feature fusion.

### 3.1. Word Embedding Module

Given an input sentence–aspect pair, we first employ the pre-trained language model RoBERTa [10] to obtain contextually enriched embeddings. Compared to BERT [9], RoBERTa introduces several optimizations: dynamic masking, where a new mask pattern is generated at each training step instead of using a fixed one; removal of the next sentence prediction objective in favor of training on contiguous sentences up to the maximum length; and a larger vocabulary (50,265 tokens versus 30,522). These improvements lead to superior downstream performance.

The input sequences $O_{s}$ and $O_{a}$ are constructed as follows and then fed into RoBERTa:

(1) $$O_{s}=\langle s\rangle ⊕S⊕\langle /s\rangle ⊕A⊕\langle /s\rangle$$

(2) $$O_{a}=\langle s\rangle ⊕A⊕\langle /s\rangle$$

where 〈s〉 is the start-of-sentence token and 〈/s〉 is the separator token in RoBERTa.

The output of the last hidden layer of RoBERTa is used as the contextual embedding matrix $H_{s}\in R^{n\times d}$ and the aspect embedding matrix $H_{a}\in R^{m\times d}$, where *d* is the hidden dimension, and *n* and *m* are the token lengths of $O_{s}$ and $O_{a}$ after subword tokenization (note that $n\geq \tilde{n}$ and $m\geq \tilde{m}$ due to subword splitting and special tokens), respectively.

(3) $$H_{s}=\mathrm{RoBERTa}\left(O_{s}\right)$$

(4) $$H_{a}=\mathrm{RoBERTa}\left(O_{a}\right)$$

Since dependency parsing, AMR parsing, and external lexical knowledge are usually defined at the word level, while RoBERTa represents text at the subword level, we perform an alignment process during preprocessing. Specifically, each original word is first tokenized by the RoBERTa tokenizer, and the correspondence between the generated subword tokens and the original word is recorded. Dependency and AMR structures are first constructed over the original word-level sentence and then projected to the subword level according to this recorded correspondence. In this way, when a word is split into multiple subwords, these subwords inherit the syntactic and semantic structural information of the original word. The resulting subword-level structural information is then used together with the RoBERTa representations in the following modules.

### 3.2. Syntactic Dependency Module

To enable the model to learn syntactic information from the dependency parse tree, we construct a syntactic dependency matrix using the LAL-Parser [31]. The process is illustrated in the left part of Figure 2.

First, the sentence *S* is parsed offline by the LAL-Parser to obtain a dependency parse tree, which is then converted into a syntactic dependency matrix. After word-to-subword alignment, the syntactic matrix is expanded to $M_{syn}\in R^{n\times n}$, representing the token-to-token syntactic connections, where *n* is the sequence length. In our implementation, the syntactic matrix is constructed as a weighted adjacency matrix based on the dependency information produced by LAL-Parser. Self-loops are added so that each token can retain its own representation during graph propagation. Since this branch focuses on modeling syntactic connectivity between aspect terms and opinion words rather than distinguishing dependency relation types, dependency labels are not encoded as separate edge features. The dependency edges are treated as undirected connections, allowing information to be propagated in both directions between syntactically related tokens. During GCN propagation, the aggregated neighboring representations are normalized according to the adjacency degree, which mitigates scale variations caused by different node degrees. Specifically, we use row-wise degree normalization. For each token *i*, the normalization factor is computed as $d_{i}=\sum_{j=1}^{n}M_{syn,ij}+1$, and the aggregated neighbor representation is divided by $d_{i}$. This implementation follows the normalization operation used in our GCN layer.

Then, the syntactic dependency matrix and the RoBERTa embedding $H_{s}\in R^{n\times d}$ are fed into a two-layer GCN to aggregate neighborhood information. A single layer cannot adequately capture multi-hop neighborhood information, whereas deeper layers increase the number of parameters and the risk of overfitting. This design is further validated in Section 4.6. The layer-wise propagation rule is as follows:

(5) $$H_{syn}^{l}=\mathrm{ReLU}\left(D^{-1}M_{syn}H_{syn}^{l-1}W\right)$$

where D is a diagonal degree matrix with $D_{ii}=\sum_{j=1}^{n}M_{syn,ij}+1$, $W\in R^{d\times d}$ is a learnable weight matrix, $H_{syn}^{l}\in R^{n\times d}$ is the output of the *l*-th layer, and l∈{1,2} with $H_{syn}^{0}=H_{s}$.

Finally, the output of the syntactic dependency module is taken from the second GCN layer:

(6) $$O_{syn}=H_{syn}^{2}$$

### 3.3. Abstract Meaning Module

Unlike dependency trees, Abstract Meaning Representation (AMR) does not rely on grammatical rules but parses a sentence into a rooted, directed semantic graph that captures semantic relations among concepts. In our preprocessing stage, the input sentence is parsed offline by the SPRING parser [32] to obtain an AMR graph. Each AMR relation is represented as a triple consisting of a source node index, a relation label, and a target node index. After aligning AMR nodes with the token sequence, these triples are converted into a token-level AMR edge-label matrix. It should be noted that we do not perform AMR parsing and AMR graph-to-token alignment from scratch. Instead, following APARN [17], we use the preprocessed AMR-derived semantic structures released with the APARN dataset. In this preprocessed data, each sentence has been associated with an AMR-based semantic relation representation, where the AMR parsing, concept-to-token alignment, and graph-to-token conversion have already been completed according to the preprocessing procedure of APARN. Therefore, our semantic module focuses on incorporating the provided AMR-derived semantic information into the proposed model rather than constructing AMR graphs from raw sentences.

Since RoBERTa operates on subword tokens, we further project the AMR structure to the RoBERTa subword level. Specifically, each original token is tokenized into RoBERTa subwords, and a subword-to-original-token mapping is recorded during preprocessing. Based on this mapping, the token-level AMR edge-label matrix is projected to the subword level, where the relation between two subwords is inherited from the AMR relation between their corresponding original tokens. Therefore, if an original token is split into multiple subwords, these subwords share the same AMR structural neighborhood of the original token. For token pairs without an explicit AMR relation, we assign a special none relation label, indicating the absence of a direct AMR semantic edge. For diagonal entries, we assign a self relation label so that each token can preserve its own semantic identity. Therefore, each matrix entry represents either a specific AMR relation label, the none label, or the self label.

After this alignment, we obtain a subword-level AMR edge-label matrix, which is padded to the maximum sequence length and aligned with the RoBERTa input sequence, including the special tokens. The discrete AMR relation labels are then mapped into dense relation embeddings. Specifically, all AMR relation labels appearing in the preprocessed data, together with special labels such as none and self, are included in the AMR relation-label vocabulary. The corresponding AMR relation embeddings are implemented as a trainable embedding table. Before training, this embedding table is initialized using Xavier uniform initialization. During supervised training, the AMR relation embeddings are updated end-to-end through back-propagation under the final sentiment classification objective, and they are not frozen during training. Formally, the embedded semantic relation tensor is denoted as $M_{sem}\in R^{n\times n\times d}$, where *n* denotes the maximum RoBERTa sequence length and *d* is the hidden dimension. Each element $M_{sem}\left[i,j,:\right]\in R^{d}$ represents the embedded AMR relation between the *i*-th and the *j*-th RoBERTa tokens. Compared with a two-dimensional adjacency matrix, this three-dimensional tensor preserves richer relation-level semantic information between token pairs. The process is illustrated in the right part of Figure 2. To inject these structural semantic priors into the token representations, we employ an axial attention layer followed by a semantic-aware attention layer.

#### 3.3.1. Axial Attention

Given the AMR-based semantic relation tensor $M_{sem}$, the axial attention layer aims to aggregate semantic relation information along two token axes. Specifically, the row axis captures the outgoing semantic relations from a token to other tokens, while the column axis captures the incoming semantic relations from other tokens to the current token.

For the row axis, we compute the attention weight between the *i*-th token and the *j*-th token as follows:

(7) $$\alpha_{ij}=\frac{\exp\left(uM_{sem}\left[i,j,:\right]\right)}{\sum_{k=1}^{n}\exp\left(uM_{sem}\left[i,k,:\right]\right)}$$

where $u\in R^{d}$ is a learnable row vector.

Then, the row-axis semantic representation of the *i*-th token $R_{i}\in R^{d}$ is obtained by the following:

(8) $$R_{i}=\sum_{j=1}^{n}\alpha_{ij}M_{sem}\left[i,j,:\right]$$

Similarly, for the column axis, the attention weight is computed as follows:

(9) $$\beta_{ji}=\frac{\exp\left(uM_{sem}\left[j,i,:\right]\right)}{\sum_{k=1}^{n}\exp\left(uM_{sem}\left[k,i,:\right]\right)}$$

The column-axis semantic representation of the *i*-th token $C_{i}\in R^{d}$ is then obtained by the following:

(10) $$C_{i}=\sum_{j=1}^{n}\beta_{ji}M_{sem}\left[j,i,:\right]$$

The two axis-level semantic representations are fused to obtain the AMR-enhanced semantic representation:

(11) $$H_{\mathrm{sem},i}=\mathrm{LayerNorm}\left(R_{i}+C_{i}\right)$$

By stacking all token representations, we obtain the following:

(12) $$H_{\mathrm{sem}}=\left[H_{\mathrm{sem},1};H_{\mathrm{sem},2};\cdots ;H_{\mathrm{sem},n}\right]\in R^{n\times d}$$

Next, we compute a query and a key to measure the semantic relevance between contextual token representations and the aggregated AMR semantic representations. The query $Q_{\mathrm{axial}}\in R^{n\times d}$ is derived from the sentence features, while the key $K_{\mathrm{axial}}\in R^{n\times d}$ encodes the axis-aggregated semantic information:

(13) $$Q_{\mathrm{axial}}=H_{s}W_{H}$$

(14) $$K_{\mathrm{axial}}=H_{\mathrm{sem}}W_{M}$$

where $W_{H}\in R^{d\times d}$ and $W_{M}\in R^{d\times d}$ are learnable weight matrices.

The scaled dot-product attention score between the contextual sentence representation and the AMR-enhanced semantic representation is then computed and normalized:

(15) $$A_{\mathrm{axial}}=\mathrm{softmax}\left(\frac{Q_{\mathrm{axial}}K_{\mathrm{axial}}^{T}}{\sqrt{d}}\right)$$

The resulting matrix $A_{\mathrm{axial}}\in R^{n\times n}$ encodes the AMR-guided semantic relevance between token pairs. This matrix serves as an additive bias that guides the subsequent semantic-aware attention.

#### 3.3.2. Semantic-Aware Attention

We feed the same sentence representations into a multi-head self-attention module enhanced with the graph-derived bias. Concretely, let *h* be the number of attention heads and $d_{h}=d/h$ be the dimension of each head. For the *i*-th head (i=1,…,h), the queries, keys, and values are obtained by projecting the embedding matrix $H_{s}$ with head-specific weight matrices:

(16) $$Q_{i}=H_{s}W_{Q,i},\;K_{i}=H_{s}W_{K,i},\;V_{i}=H_{s}W_{V,i}$$

where $W_{Q,i},W_{K,i},W_{V,i}\in R^{d\times d_{h}}$ are learnable parameters.

For each head, the scaled dot-product attention scores are computed, and the shared axial bias $A_{\mathrm{axial}}$ is added before the softmax normalization:

(17) $${\mathrm{head}}_{i}=\mathrm{softmax}\left(\frac{Q_{i}K_{i}^{T}}{\sqrt{d_{h}}}+A_{\mathrm{axial}}\right)V_{i}$$

The outputs of all heads are first concatenated along the feature dimension to form a unified representation $O_{\mathrm{concat}}\in R^{n\times d}$:

(18) $$O_{\mathrm{concat}}=\mathrm{Concat}\left({\mathrm{head}}_{1},\ldots ,{\mathrm{head}}_{h}\right)$$

Subsequently, this concatenated representation is linearly projected using a learnable weight matrix $W_{O}\in R^{d\times d}$ to obtain the final output of the semantic module $O_{\mathrm{sem}}\in R^{n\times d}$:

(19) $$O_{\mathrm{sem}}=O_{\mathrm{concat}}W_{O}$$

Thus, the semantic module yields a contextualized representation $O_{\mathrm{sem}}$ that is enriched with abstract structural relations between concepts.

### 3.4. Knowledge Enhancement Module

To enrich the semantic features, we incorporate external knowledge from WordNet [22], a large-scale lexical database that organizes English words into sets of synonyms, known as synsets. WordNet encodes semantic relations such as hypernymy, hyponymy, meronymy, and antonymy and covers approximately 166,000 word–meaning pairs. Knowledge graph embeddings are trained on WordNet using DistMult via OpenKE [33], yielding a frozen embedding table $E\in R^{V\times d_{k}}$, where *V* is the vocabulary size and $d_{k}$ is the knowledge embedding dimension.

Specifically, we adopt a vocabulary-level alignment strategy that is consistent with RoBERTa byte-level BPE tokenization. For each RoBERTa vocabulary item, we first normalize the token by removing byte-level markers such as “Ġ” and “Ċ”, converting it to lowercase, and removing possible subword prefixes. We then generate candidate lexical forms by normalizing separators, e.g., replacing hyphens with underscores or converting underscores back to spaces. These candidates are matched against the WordNet lexical-form entities. If a candidate lexical form is found in the WordNet entity vocabulary, the corresponding knowledge graph embedding is assigned to the RoBERTa token id. Therefore, during model execution, the same RoBERTa token ids used by the text encoder can be directly used to retrieve the corresponding knowledge vectors.

When one lexical form corresponds to multiple WordNet synsets, we do not perform explicit word sense disambiguation or manually select a single synset. Instead, the lexical form itself is represented as an independent lemma entity in the WordNet knowledge graph and is connected to all its candidate synsets through lemma–synset relations. The vector assigned to a matched RoBERTa token is therefore the learned embedding of this lexical-form entity, which encodes information from all related synsets and lexical relations.

For RoBERTa subword tokens that cannot be matched to WordNet lexical entries, their knowledge vectors are randomly initialized from U(−0.25,0.25). We further compute the dataset-level matching ratio, defined as the proportion of RoBERTa subword tokens whose normalized lexical forms can be matched to WordNet lexical-form entities. The matching ratios are 36.53%, 32.57%, and 29.98% on the Laptop, Restaurant, and Twitter datasets, respectively.

For multi-word aspects, the aspect phrase is tokenized by the RoBERTa tokenizer, and each aspect token retrieves its knowledge embedding independently. The model then computes attention between the knowledge-enhanced aspect token sequence and the knowledge-enhanced sentence token sequence, allowing multi-word aspects to be handled as token sequences. The contextual RoBERTa representations further help the downstream attention and classification layers select useful knowledge signals according to the sentence context.

Following the input construction in the word embedding module, $O_{s}$ and $O_{a}$ denote the RoBERTa token-id sequences of the sentence–aspect pair and the aspect, respectively. For each token id in these sequences, its knowledge embedding is retrieved from the frozen knowledge embedding table:

(20) $$E_{s}=\left[E\left[o_{1}^{s}\right];E\left[o_{2}^{s}\right];\ldots ;E\left[o_{n}^{s}\right]\right]\in R^{n\times d_{k}}$$

(21) $$E_{a}=\left[E\left[o_{1}^{a}\right];E\left[o_{2}^{a}\right];\ldots ;E\left[o_{m}^{a}\right]\right]\in R^{m\times d_{k}}$$

To enhance the knowledge representations with contextual semantics, we concatenate the knowledge embeddings with RoBERTa-encoded representations along the feature dimension:

(22) $$S_{\mathrm{know}}=\mathrm{Concat}\left(E_{s},H_{s}\right)$$

(23) $$A_{\mathrm{know}}=\mathrm{Concat}\left(E_{a},H_{a}\right)$$

Here, $S_{\mathrm{know}}\in R^{n\times (d_{k}+d)}$ and $A_{\mathrm{know}}\in R^{m\times (d_{k}+d)}$.

To capture the semantic correlation between the sentence and the aspect in the knowledge-enhanced space, we employ a simplified attention mechanism. Specifically, we compute the dot-product attention between aspect-enhanced and sentence-enhanced knowledge representations.

(24) $$\mathrm{score}=\mathrm{softmax}(A_{\mathrm{know}}S_{\mathrm{know}}^{T})$$

The resulting score matrix $\mathrm{score}\in R^{m\times n}$ is then used to aggregate the sentence features, yielding a matrix $O_{\mathrm{know}}\in R^{m\times d}$ in which each row corresponds to an aspect token attended over the sentence:

(25) $$O_{\mathrm{know}}=\mathrm{score}\;H_{s}$$

Through the three parallel branches described above, the model obtains feature representations from syntactic, semantic, and knowledge dimensions. All branches operate on the RoBERTa embeddings and extract complementary information, enabling the model to capture multi-view characteristics.

### 3.5. Feature Fusion Module

**Figure 3:**
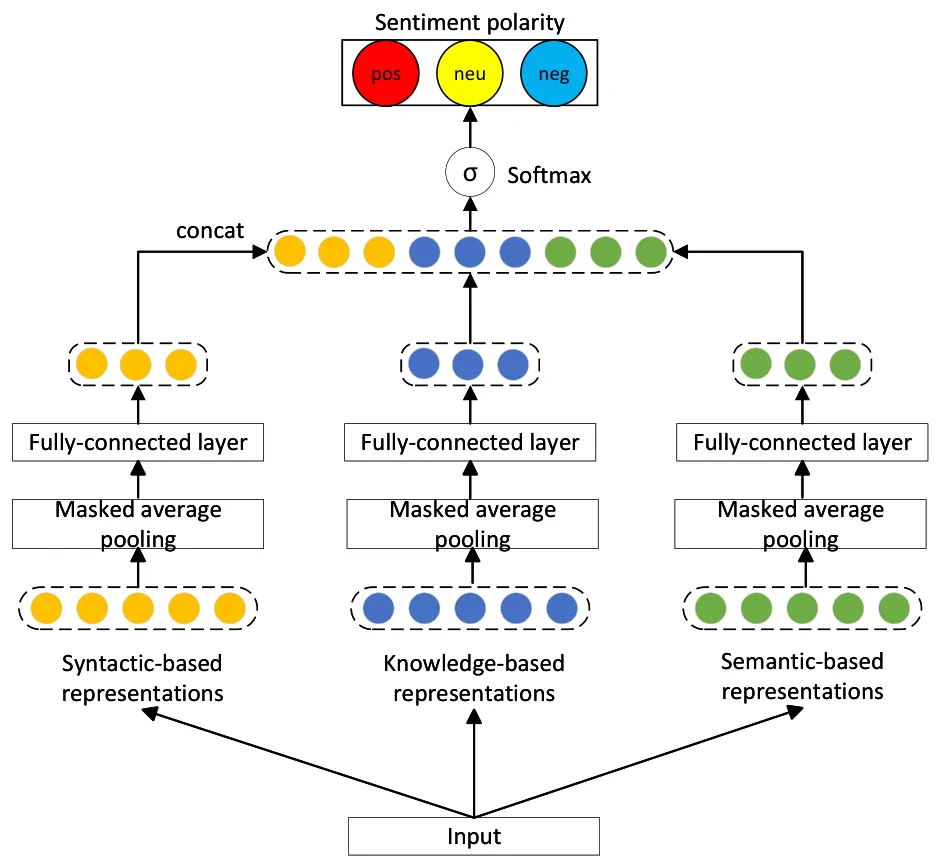
The architecture of the feature fusion module.

The three outputs $O_{syn}$, $O_{sem}$, and $O_{know}$ are fused in the feature fusion module, as shown in Figure 3.

Although $O_{syn}$, $O_{sem}$, and $O_{know}$ are obtained from heterogeneous branches and may have different sequence lengths or feature dimensions, they are first converted into fixed-dimensional aspect-level representations before fusion. Specifically, let $O_{r}\in R^{L_{r}\times d_{r}}$ denote the output of branch r∈{syn,sem,know}, where $L_{r}$ is the corresponding sequence length and $d_{r}$ is the feature dimension. For the syntax and semantic branches, $L_{r}=n$, where *n* is the RoBERTa subword sequence length. For the knowledge branch, $L_{r}=m$, where *m* is the number of valid aspect tokens. An aspect mask $m_{r}^{asp}\in {\left\{0,1\right\}}^{L_{r}}$ is used to select the target-aspect positions. For the syntax and semantic branches, $m_{r,i}^{asp}=1$ if the *i*-th RoBERTa subword belongs to the target aspect and 0 otherwise. For the knowledge branch, since $O_{know}$ is already constructed over the aspect tokens, $m_{know,i}^{asp}=1$ for valid aspect-token positions and 0 for padded positions. The aspect-level representation of each branch is obtained by masked average pooling:

(26) $$o_{r}=\frac{\sum_{i=1}^{L_{r}}m_{r,i}^{asp}O_{r,i}}{\sum_{i=1}^{L_{r}}m_{r,i}^{asp}},\;r\in \left\{syn,sem,know\right\},$$

where $o_{r}\in R^{d_{r}}$ denotes the aggregated aspect-level vector of the corresponding branch. This operation converts sequence-level branch outputs into fixed-dimensional aspect-level vectors.

Since these features have different dimensionalities, each is first passed through a fully connected layer to project it into a unified dimensional space and then concatenated to form $O_{final}\in R^{3d_{f}}$, where $d_{f}$ denotes the unified feature dimension after projection.

(27) $$O_{syn\_o}={\mathrm{FC}}_{syn}\left(o_{syn}\right),\;O_{sem\_o}={\mathrm{FC}}_{sem}\left(o_{sem}\right),\;O_{know\_o}={\mathrm{FC}}_{know}\left(o_{know}\right)$$

where $O_{syn\_o}$, $O_{sem\_o}$, and $O_{know\_o}$ are projected into the same dimensional space $R^{d_{f}}$, enabling direct fusion of heterogeneous aspect-level representations.

(28) $$O_{final}=\mathrm{Concat}\left(O_{syn\_o},O_{sem\_o},O_{know\_o}\right)$$

The fused representation $O_{final}$ integrates complementary information from syntactic dependency structures, AMR-based abstract semantics, and external lexical knowledge, forming a unified aspect-level representation for sentiment classification.

Finally, the fused aspect-level representation is passed through a linear projection layer with softmax activation for sentiment prediction:

(29) $$P=\mathrm{softmax}(W_{final}O_{final}+b_{final})$$

where $W_{final}\in R^{C\times 3d_{f}}$ and $b_{final}\in R^{C}$ are learnable parameters, and *C* is the number of sentiment classes (typically C=3 for positive, neutral, and negative). The output $P\in R^{1\times C}$ is the predicted probability distribution over sentiment classes.

### 3.6. Training

We employ the cross-entropy loss with $L_{2}$ regularization for parameter optimization:

(30) $$L=-\sum_{i=1}^{D}\sum_{c=1}^{C}y_{i,c}\log P_{i,c}+\lambda {∥\theta ∥}^{2}$$

where *D* is the size of the training set, $P_{i,c}$ denotes the predicted probability of the *c*-th sentiment class for the *i*-th instance, $y_{i,c}$ is the corresponding one-hot ground-truth label, θ denotes all trainable parameters, and λ is the regularization coefficient.

## 4. Results

### 4.1. Datasets

We conduct experiments on three public benchmark datasets: Laptop14, Restaurant14, and Twitter. Laptop14 and Restaurant14 originate from SemEval-2014 Task 4 [2], while the Twitter dataset is a collection of tweets [34]. Laptop14 contains over 3000 laptop review sentences, Restaurant14 contains over 3000 restaurant review sentences, and Twitter contains over 7000 tweets about products, celebrities, and companies. The statistics of the datasets are summarized in Table 2.

### 4.2. Parameter Settings

In the word embedding module, we use RoBERTa-base with a learning rate of 10^−5^. The syntactic dependency module uses a two-layer GCN with a dropout rate of 0.1. The abstract semantic module employs eight attention heads. The hidden-state dimension is set to 768 to maintain dimensional consistency across modules. The knowledge embedding dimension is set to 200. We use the Adam optimizer and a batch size of 16. AMR graphs are parsed and stored before model training, and WordNet embeddings are pre-trained offline and then directly loaded during training and inference. Training is conducted on an NVIDIA V100-32G GPU with an Intel Xeon Gold 6130 CPU, PyTorch 1.8.1, Python 3.8, and CUDA 11.1.

To improve the transparency of computational cost, we report the computational profile of KEMFF under a fixed hardware setting. The reported statistics include the number of trainable parameters, preprocessing/loading time, average training time per epoch, total training time, inference time per sample, peak GPU memory consumption, offline processing time, and memory consumption of LAL-Parser and WordNet. The results are shown in Table 3. All results are measured on the same hardware setting. “Preprocess/Load” includes dataset loading, loading of preprocessed dependencies and AMR-based graphs, knowledge embedding loading, tokenizer construction, and model initialization. “Train/Epoch” denotes the average training-validation time per epoch. “Inference” denotes the average inference latency per test sample. “Peak Memory” is measured by the maximum allocated GPU memory during training using torch.cuda.max_memory_allocated. “OF-LP” records the running seconds and peak memory consumption of LAL-Parser. “OF-WN” records the running minutes and peak memory consumption of WordNet. Note that “OF-WN” is dataset-independent and thus reported only once.

### 4.3. Comparison Methods

We compare KEMFF with the following recent and competitive baselines:

1. AOA [35]: An attention-over-attention model that jointly models aspect terms and text to build aspect-context connections.
2. AEN_BERT [36]: An attention encoder network combined with BERT for target-dependent sentiment classification.
3. ASGCN [5]: A GCN-based model that introduces A-links and S-links to better capture structural and aspect-related relations.
4. BERT_SPC [9]: A BERT-based sentence-pair classification model specifically designed for target sentiment tasks.
5. RoBERTa_SPC: We replace the BERT encoder in BERT_SPC with the RoBERTa backbone. Therefore, it serves as a matched RoBERTa-only control model for examining whether the gains of KEMFF come from the proposed fusion architecture rather than from the RoBERTa encoder alone.
6. LCF_BERT [37]: A local context focus mechanism that enhances BERT for ABSA.
7. DualGCN [6]: A dual-GCN architecture that separately processes syntactic and semantic features.
8. SSEGCN [38]: A masking-enhanced GCN that improves the capture of aspect-context relationships.
9. DotGCN [39]: A discrete latent tree model that replaces syntactic dependency trees for ABSA.
10. APARN [17]: An AMR-based path aggregation relational network for ABSA.
11. RoBERTa-ACOP and RoBERTa-SDACP [40]: Multi-task training that combines aspect-context order prediction and syntactic dependency prediction with sentiment classification.
12. Semantic-HGCN [41]: A semantic heterogeneous GCN with global attention for ABSA.
13. PHNN [42]: A prompt-enhanced hybrid neural network that fuses CNNs and GCNs to improve semantic modeling.
14. RDGCN [13]: A reinforced dependency GCN that uses reinforcement learning to weight syntactic dependencies.
15. TextGT [12]: A dual-view graph transformer that processes text data to enhance semantic representations.

All baseline results have been reproduced from their original implementations. For AOA, AEN_BERT, ASGCN, BERT_SPC, and LCF_BERT, we use the ABSA-PyTorch Implementation (https://github.com/songyouwei/ABSA-PyTorch (accessed on 1 February 2026)). RoBERTa_SPC is implemented by replacing the BERT encoder in BERT_SPC with RoBERTa-base while keeping the original sentence-pair classification setting unchanged. For other models, we obtain the source code from the corresponding publications or official repositories. All experiments are conducted using the original code and hyperparameters specified in the corresponding papers. For all reproduced baselines and KEMFF, we used the same official train/test splits of Laptop14, Restaurant14, and Twitter as summarized in Table 2. The official training set was used for model training, and no test instance was used for gradient-based parameter updating. To ensure fair comparison and reproducibility, all models were run under the same random seed protocol. For each reproduced baseline, we kept the original preprocessing pipeline, input construction strategy, hyperparameter settings, and checkpoint-selection strategy provided by its released implementation. All models were evaluated on the same official train/test splits using the same evaluation metrics, and a consistent reporting protocol was applied to KEMFF and all reproduced baselines to ensure comparability.

### 4.4. Main Results

Table 4 reports the best single-run results of KEMFF and the baselines on the three datasets, with the best results marked in bold and the second-best results underlined. These results highlight the maximum accuracy and macro-F1 scores achieved during our experiments and serve as a direct performance comparison against existing methods.

As shown in Table 4, KEMFF achieves the best performance among the compared baselines on Laptop14 and Restaurant14 and obtains competitive results on Twitter. In detail, on Laptop14, KEMFF surpasses the strongest compared baseline by 1.64% in Accuracy and 1.51% in Macro-F1. On Restaurant14, KEMFF improves over the strongest compared baseline by 0.98% in Accuracy and 1.22% in Macro-F1. On Twitter, although KEMFF does not achieve the highest Accuracy or Macro-F1, its performance remains competitive, with only 0.89% and 0.16% gaps from the best compared baseline, respectively. These results confirm that the multidimensional feature fusion with knowledge enhancement improves over single-stream or dual-stream models. Furthermore, models built upon pre-trained language models consistently outperform those without, as the rich contextual representations learned from large-scale unlabeled data significantly benefit downstream fine-tuning.

Notably, syntax-based models (DualGCN, SSEGCN) show substantial accuracy improvements over the context-only BERT_SPC on Laptop14 (+4.71%, +4.52%) and Restaurant14 (+1.95%, +2.66%), but only marginal gains on Twitter (+0.2%, +1.02%). This may be because Laptop14 and Restaurant14 contain a higher proportion of multi-aspect sentences (26.58% and 20.05%, respectively), where syntactic dependency trees effectively resolve long-distance aspect-opinion relations through their non-local modeling capacity, whereas Twitter reviews are largely informal and less sensitive to syntactic structure.

For the potential encoder-backbone confound, KEMFF consistently outperforms RoBERTa_SPC on all three datasets. Specifically, KEMFF improves Accuracy/Macro-F1 over RoBERTa_SPC by 4.39%/5.42%, 3.31%/5.45%, and 2.53%/3.00% on Laptop14, Restaurant14, and Twitter, respectively. Since both models use RoBERTa-base as the backbone encoder, these results indicate that the performance gains of KEMFF are not merely due to the pre-trained encoder, but they mainly come from the proposed fusion of syntactic dependency features, AMR-based semantic relations, and WordNet-based lexical knowledge.

### 4.5. Ablation Study

To verify the effectiveness of different components in KEMFF, we conduct a series of ablation experiments on Laptop14, Restaurant14, and Twitter. The proposed model consists of three major branches: the syntactic dependency branch, the abstract meaning branch, and the knowledge enhancement branch. For simplicity, we denote them as S, A, and K, respectively. Specifically, S represents the syntactic dependency model based on dependency parsing and GCN, A represents the AMR-based abstract meaning model with axial attention and semantic-aware attention, K represents the WordNet-based knowledge enhancement model, and w/o means without the corresponding model. The results are presented in Table 5.

As shown in Table 5, removing any branch from the full model leads to a decrease in performance, indicating that syntactic dependency information, AMR-based semantic information, and external knowledge all contribute to ABSA. Specifically, when the syntactic branch is removed, the performance drops on all three datasets, showing that dependency relations are useful for capturing aspect-opinion connections. When the semantic branch is removed, the model also suffers from performance degradation, suggesting that AMR provides complementary semantic information beyond surface syntax. When the knowledge enhancement branch is removed, the model suffers from the largest performance degradation, especially on Laptop14 (−3.17% accuracy, −4.1% macro-F1) and Restaurant14 (−1.52% accuracy, −3.11% macro-F1), indicating that in grammatically well-structured and syntax-sensitive datasets, external knowledge greatly benefits the construction of syntactic and semantic features.

The single-branch variants further reveal the different contributions of the three information sources. w/o S + A retains only the knowledge branch, w/o S + K retains only the AMR-based semantic branch, and w/o A + K retains only the syntactic branch. Among them, w/o A + K generally obtains the weakest performance, showing that syntactic dependency information alone is insufficient for robust aspect-level sentiment classification. In contrast, the knowledge-only variant w/o S + A performs relatively better than the other single-branch variants, indicating that external lexical knowledge provides useful sentiment-related cues. Nevertheless, all single-branch variants are substantially inferior to the full model, confirming that syntax, semantics, and external knowledge are complementary and should be jointly modeled.

Overall, the ablation results demonstrate that all three components contribute positively to the final prediction. The two-branch variants generally outperform the single-branch variants, while the full KEMFF model consistently achieves the best results. This confirms that syntactic dependency information, AMR-based semantic information, and external knowledge are complementary rather than redundant, and it validates the effectiveness of the proposed multidimensional feature fusion framework.

### 4.6. Fine-Grained Ablation Study

The branch-level ablation study in Table 5 verifies the overall contribution of the syntactic, semantic, and knowledge branches. However, it does not isolate several internal design choices of KEMFF. Therefore, we further conduct a fine-grained ablation study to evaluate the effects of axial attention and the feature fusion module. The results are reported in Table 6. In particular, “w/o at” removes the axial attention bias $A_{\mathrm{axial}}$ from the semantic-aware attention layer, reducing it to standard multi-head self-attention over RoBERTa representations. This variant evaluates whether the axial attention is useful for guiding token-level semantic interaction.“w/o ff” first projects the three branch representations into the same dimensional space and then averages them instead of concatenating them. This variant evaluates whether the proposed feature fusion module is more effective than simpler fusion strategies.

As shown in Table 6, removing axial attention leads to a performance decrease on all three datasets. This confirms that axial attention is not only useful as an independent representation, but it is also beneficial when used as an attention bias to guide token-level semantic interaction. Compared with standard self-attention, the AMR-guided semantic-aware attention can better emphasize token pairs connected by abstract semantic relations.

For feature fusion, average fusion performs worse than the proposed fusion module. This is because average fusion forces different branches to contribute equally and may weaken branch-specific complementary information. In contrast, the proposed projection-and-concatenation strategy first maps syntax, semantics, and knowledge features into a unified latent space and then preserves their complementary signals through concatenation. These results validate the effectiveness of the proposed feature fusion module.

### 4.7. Stability Analysis

To further evaluate the robustness of the proposed model and reduce the influence of random initialization, we conduct repeated experiments with five different random seeds. In addition to KEMFF, four strong baseline models, including syntax-based modeling, knowledge-enhanced modeling, hybrid-based modeling, and transformer/graph-based modeling, namely, DualGCN, APARN, PHNN, and TextGT, are included for comparison. For each model, we report the mean and standard deviation of Accuracy and Macro-F1 on Restaurant14, Laptop14, and Twitter. To further examine whether the observed improvements are statistically reliable, we conduct paired two-tailed Student’s *t*-tests between KEMFF and the strongest baseline model on each dataset under the same five random seeds. Since KEMFF and each compared baseline are evaluated under identical random seeds, the results form paired observations, making the paired test appropriate for this setting. Following common practice, differences are considered statistically significant when p<0.05. The results are shown in Table 7.

As shown in Table 7, KEMFF achieves the best average performance on Laptop14 and Restaurant14 among the compared models. On Laptop14, KEMFF obtains an average Accuracy of 82.81±0.54 and an average Macro-F1 of 79.62±0.97, outperforming the other baseline models in both metrics. On Restaurant14, KEMFF achieves an average Accuracy of 87.77±0.42 and an average Macro-F1 of 82.30±0.83, also ranking first among the compared methods. The paired two-tailed Student’s *t*-tests further confirm the statistical reliability of these improvements. Specifically, the *p*-values are 0.0024 for Laptop14 Accuracy, 0.0309 for Laptop14 Macro-F1, 0.0191 for Restaurant14 Accuracy, and 0.0265 for Restaurant14 Macro-F1. Since all these values are below 0.05, the improvements of KEMFF on Laptop14 and Restaurant14 are statistically significant. These results indicate that the proposed syntax–semantics–knowledge fusion framework can maintain stable and strong performance across different random initializations.

On Twitter, KEMFF obtains an average Accuracy of 75.35±1.00 and an average Macro-F1 of 74.66±1.33. Although KEMFF does not achieve the highest average result on this dataset, its performance remains competitive with TextGT, which achieves the best average scores on Twitter. Since KEMFF does not outperform the strongest baseline on Twitter, no significance marker is reported for this dataset. This observation is consistent with the main experimental results and suggests that Twitter is more challenging due to its informal expressions, noisy syntax, and diverse linguistic patterns. Such characteristics may reduce the effectiveness of dependency-based and AMR-based structural information.

It should be noted that the main comparison table reports the best single-run results, while Table 7 reports the mean and standard deviation over five runs. Therefore, the average results in Table 7 may be slightly lower than the best results reported in the main comparison table. Overall, the stability analysis demonstrates that the performance gains of KEMFF on Restaurant14 and Laptop14 are not caused by a single favorable run, and the relatively small standard deviations further indicate that the proposed model is stable under different random seeds. Together with the significance tests, these results provide stronger statistical evidence for the robustness and effectiveness of KEMFF.

### 4.8. Effect of External Knowledge

To compare different external knowledge sources fairly, we construct WordNet-, SentiWordNet-, and SenticNet-based knowledge embeddings using the same pipeline. For each source, we first convert the original resource into a set of triples, then train knowledge graph embeddings using DistMult through OpenKE, and finally align the learned entity embeddings with the RoBERTa vocabulary to obtain a token-indexed knowledge embedding table.

For WordNet, synsets and lemmas are treated as entities, and lexical-semantic relations such as hypernymy, hyponymy, meronymy, holonymy, synonymy, antonymy, and similar-to relations are extracted. For SentiWordNet, we additionally encode sentiment labels and discretized positive, negative, and objective scores, while preserving lemma-synset and selected WordNet semantic relations. For SenticNet, each concept is treated as an entity, and triples are constructed from semantic associations, polarity labels, polarity-value buckets, primary and secondary moods, and sentic dimensions such as pleasantness, attention, sensitivity, and aptitude. All three knowledge sources are embedded using the same KGE model DistMult and aligned to the same RoBERTa vocabulary. Therefore, the comparison focuses on the effect of different knowledge sources rather than differences in the embedding or alignment procedure.

Furthermore, to verify whether the gains come from genuine external knowledge rather than simply additional parameters or input dimensions, we add a random-knowledge control setting and a shuffled-knowledge control setting. The random setting uses a frozen random embedding table with the same size and dimensionality as the WordNet embedding table initialized from U(−0.25,0.25). The comparison with this control setting helps demonstrate whether structured external knowledge contributes beyond random features. The shuffled-knowledge setting randomly permutes the WordNet embedding table across RoBERTa token ids. In this way, the embedding distribution and norm statistics of WordNet are preserved, but the correct correspondence between lexical tokens and WordNet knowledge is destroyed. Finally, the experimental results are shown in Table 8.

As shown in Table 8, WordNet, SentiWordNet, and SenticNet all outperform the random-knowledge control, indicating that structured external knowledge is beneficial. The random-knowledge control obtains the lowest performance on all three datasets, indicating that the improvement of KEMFF is not simply caused by additional parameters or increased input dimensionality. In contrast, structured knowledge sources generally bring clear performance gains, confirming that external knowledge provides useful complementary information for ABSA. However, the effectiveness of different knowledge sources is dataset-dependent. SenticNet achieves the best results on Laptop14, with 83.97% accuracy and 81.27% Macro-F1, suggesting that sentiment- and commonsense-oriented knowledge may be helpful for product-review scenarios. WordNet performs best on Restaurant14 and Twitter, achieving 88.29% accuracy and 83.19% Macro-F1 on Restaurant14 and 76.37% accuracy and 76.02% Macro-F1 on Twitter, showing more stable cross-domain effectiveness in our experiments. Although SentiWordNet and SenticNet contain explicit sentiment-related information, they do not consistently outperform WordNet, possibly because sentiment polarity in ABSA is highly aspect- and context-dependent, and sentiment-oriented resources may introduce noisy or domain-specific affective associations. Therefore, we adopt WordNet as the default external knowledge source in KEMFF, while the comparison also indicates that other structured knowledge resources may be beneficial in specific domains.

We also observe that Shuffled Knowledge consistently outperforms Random Knowledge on all three datasets, improving accuracy by 1.41%, 5.34%, and 4.74% on Laptop14, Restaurant14, and Twitter, respectively, and improving Macro-F1 by 0.18%, 5.26%, and 2.31%. This suggests that trained WordNet embeddings are less harmful than frozen random vectors even when their lexical alignment is disrupted. However, Shuffled Knowledge is still clearly inferior to the correctly aligned WordNet setting. Compared with Shuffled Knowledge, WordNet improves accuracy by 4.76%, 4.46%, and 8.62% on Laptop14, Restaurant14, and Twitter, respectively, with corresponding Macro-F1 gains of 5.15%, 4.39%, and 11.16%. These results indicate that preserving the embedding distribution alone is insufficient, and that correct token–knowledge correspondence and structured lexical-semantic relations are crucial for achieving the full performance gain.

### 4.9. Effect of GCN Depth

**Figure 4:**
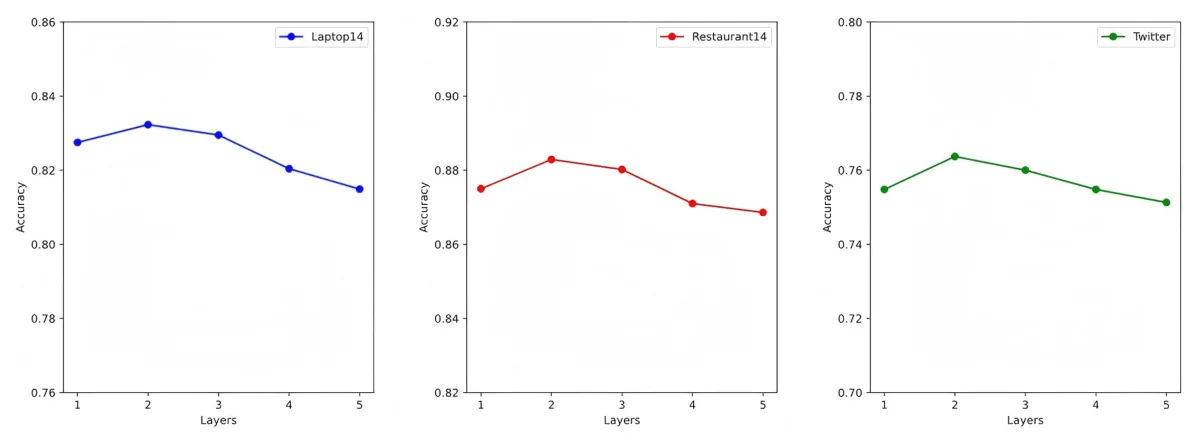
Effect of GCN depth on accuracy for Laptop14, Restaurant14, and Twitter.

We further investigate the impact of the number of GCN layers on the three datasets, as shown in Figure 4. The depth of GCN controls the range of syntactic information aggregation over the dependency graph. A one-layer GCN captures only direct syntactic neighbors, while deeper GCNs enable tokens to receive information from multi-hop syntactic contexts. Therefore, the number of GCN layers needs to be carefully selected to balance useful syntactic propagation and noise accumulation.

As shown in Figure 4, the best overall performance is achieved when the number of GCN layers is set to 2. This suggests that two-hop syntactic information is sufficient for modeling most aspect–opinion associations in ABSA. Compared with a single-layer GCN, a two-layer GCN can capture more informative dependency paths between aspect terms and opinion expressions, especially when they are not directly connected in the dependency tree.

However, stacking more than two GCN layers does not further improve performance. One possible reason is that excessive graph propagation may introduce irrelevant information from distant nodes, thereby weakening aspect-specific sentiment representations. In addition, deeper GCNs may suffer from over-smoothing, where token representations become less distinguishable after repeated propagation. They also introduce more parameters, which may increase the risk of overfitting on relatively small ABSA datasets. Therefore, we set the number of GCN layers to 2 in KEMFF.

### 4.10. Case Study

To qualitatively evaluate KEMFF, we compare its predictions with those of AOA (non-pretrained), BERT_SPC (pre-trained), and SSEGCN (non-knowledge-enhanced) on five selected sentences covering multiple aspects and challenging linguistic phenomena. The results are listed in Table 9, where P, N, and O denote positive, negative, and neutral, respectively, and correct predictions are in bold.

In the first sentence, which contains two aspects with opposite sentiments, the pre-trained models outperform AOA. The second and third sentences require the detection of keywords such as “biggest” and “not’’, which demand semantic understanding, and the pre-trained methods handle them correctly. The fourth sentence lacks an explicit sentiment expression; AOA and BERT_SPC fail to capture the negation “not’’ from the syntactic dimension and thus produce incorrect predictions. The last example demonstrates that KEMFF, by better integrating syntax and semantics with external knowledge, can handle more complex and informal sentences, correctly predicting both aspects as negative.

### 4.11. Error Analysis

To further understand the limitations of KEMFF in informal contexts, we conduct an error analysis on the Twitter dataset. Table 10 presents five representative error cases selected from the prediction results of KEMFF.

As shown in Table 10, one major source of errors lies in the model’s limited ability to capture implicit sentiment and pragmatic cues in informal Twitter texts. In the first case, the negative attitude toward “barack obama” is not expressed by direct negative sentiment words but through a rhetorical question and sarcastic expression such as “maybe i’ll win one next year then”. KEMFF predicts this sentence as neutral, suggesting that the model may rely more on surface-level semantic information and fail to recognize pragmatic signals such as irony, rhetorical questioning, and implicit criticism. Similarly, in the fifth case, the positive sentiment toward “oprah” is conveyed through the mild expression “should be interesting”, which indicates expectation rather than strong subjective emotion. Since this expression is weaker than explicit sentiment words such as “great” or “love”, the model incorrectly classifies it as neutral.

Another typical error type involves noisy and informal social media expressions. In the second case, the sentence about “madonna” contains clear positive cues, including “really cool”, “awesome”, and the emoticon “:)”, but KEMFF still predicts a neutral label. This indicates that informal grammar, conversational wording, and noisy sentence structures may weaken the model’s recognition of sentiment polarity. Although such expressions are common in Twitter data, they are often less regular than formal reviews and may be harder for the model to encode accurately. Therefore, the model may underestimate sentiment intensity when opinion words are embedded in colloquial or syntactically loose contexts.

The remaining errors further show that KEMFF may confuse factual semantic polarity with subjective sentiment, and may also fail to identify the key sentiment-bearing word correctly. In the third case, the sentence about “obama” is mainly a factual news-style update, but the phrase “under attack” introduces negative semantic information, causing the model to predict a negative label although the gold label is neutral. In the fourth case, the model predicts a positive label for the sentence about “taylor swift”, possibly because artist names and music-related contexts are often associated with positive preference in social media data. However, it fails to capture the crucial negative cue “saddest”. Overall, these cases suggest that Twitter sentiment classification remains challenging due to sarcasm, weak implicit sentiment, informal expressions, factual statements containing polarity-bearing words, and insufficient aspect-specific sentiment alignment.

## 5. Conclusions

In this paper, we propose KEMFF, a knowledge-enhanced and multidimensional feature fusion model for aspect-based sentiment analysis. By constructing a syntactic dependency matrix from the dependency parse tree, the model learns syntactic features of sentences. By further introducing AMR, the model transforms abstract semantic structures into a semantic relation tensor and uses attention mechanisms to capture semantic relations, thereby complementing traditional syntactic representations with richer semantic information. External knowledge from WordNet is then embedded to capture broader lexical relations, such as synonymy, antonymy, hyponymy, and meronymy, effectively enhancing syntactic and semantic representations. Finally, the syntactic, semantic, and knowledge features are fused, improving sentiment characterization. Experimental results show that KEMFF achieves strong performance on Laptop14 and Restaurant14 and remains competitive on Twitter, validating the effectiveness of the proposed multidimensional feature fusion framework.

Nonetheless, KEMFF does not achieve the highest performance on the Twitter dataset. This may be attributed to the informal and diverse nature of social media texts, where sentences often contain irregular grammar, colloquial expressions, abbreviations, emoticons, implicit opinions, and sarcasm. Such linguistic phenomena make it difficult for dependency parsers and AMR parsers to construct reliable structural representations. In addition, sentiment expressions in Twitter are often highly context-dependent and may not be explicitly associated with the target aspect through clear syntactic or semantic paths. As a result, the syntactic and AMR-based semantic information used in KEMFF may become less stable in noisy social media scenarios.

In future work, we plan to further improve the robustness of KEMFF for informal and noisy texts. One possible direction is to incorporate social-media-oriented linguistic features. Another direction is to design more flexible structure learning mechanisms that can reduce reliance on external parsers and adaptively capture aspect–opinion associations from noisy contexts. Moreover, we will explore more fine-grained knowledge selection strategies to filter irrelevant lexical knowledge and strengthen aspect-specific sentiment reasoning. These improvements may help the model better handle implicit sentiment, informal expressions, and complex pragmatic phenomena in real-world ABSA scenarios.

## Figures and Tables

**Table 1:** Comparison between KEMFF and closely related ABSA models.

Model	PLM	Syntactic Modeling	AMR/Semantic Modeling	External Knowledge	Fusion Strategy
ASGCN	GloVe	Dependency GCN	No	No	GCN output classification
DualGCN	BERT	Dependency graph	Attention-based semantic graph	No	Dual graph fusion
APARN	BERT/RoBERTa	No explicit dependency GCN	AMR paths	No	AMR path aggregation
KGAN	BERT	Syntax-aware	No AMR	WordNet	Hierarchical knowledge fusion
KA-GCN	RoBERTa	Dependency GCN	No AMR	SentiWordNet	Knowledge-aware GCN
DGGCN	BERT	Dependency GCN	No AMR	SenticNet	Dual-gated propagation
DSSK-GAN	BERT	Graph-based	No AMR	Static and dynamic knowledge	Graph augmentation
TextGT	BERT/RoBERTa	Graph view	Semantic graph	No	Graph Transformer
KEMFF	RoBERTa	Dependency GCN	AMR relation modeling with axial attention	WordNet	Aspect-level multidimensional fusion

**Table 2:** Statistics of the experimental datasets.

Dataset	Split	Positive	Negative	Neutral
Laptop14	Train	994	870	464
	Test	341	128	169
Restaurant14	Train	2164	807	637
	Test	728	196	196
Twitter	Train	1561	1560	3127
	Test	173	173	346

**Table 3:** Computational profile of KEMFF.

Dataset	Trainable Params	Preprocess/ Load	Train/ Epoch	Total Train	Inference	Peak Memory	OF-LP	OF-WN
Laptop	408.11 M	130.16 s	85.68 s	71.4 min	9.91 ms/sample	16.52 GB	190.09 s/3.53 G	45.81 min/4.07 G
Restaurant	408.11 M	194.59 s	137.21 s	110.2 min	10.01 ms/sample	16.52 GB	301.15 s/3.78 G	–
Twitter	408.11 M	242.82 s	178.39 s	148.7 min	10.69 ms/sample	16.52 GB	432.28 s/3.82 G	–

**Table 4:** Performance comparison on Laptop14, Restaurant14, and Twitter.

Model	Laptop14		Restaurant14		Twitter	
	Acc.	Macro-F1	Acc.	Macro-F1	Acc.	Macro-F1
AOA	72.41	67.06	78.84	67.24	71.10	69.26
AEN_BERT	78.53	74.39	81.16	70.85	73.70	70.92
ASGCN	73.51	69.04	80.63	71.18	70.66	68.86
BERT_SPC	76.49	71.97	84.38	77.05	76.16	75.27
RoBERTa_SPC	78.84	75.32	84.98	77.74	73.84	73.02
LCF_BERT	80.72	77.02	85.98	79.41	74.13	72.99
DualGCN	81.20	78.10	86.33	80.51	76.36	75.49
SSEGCN	81.01	77.96	87.04	81.09	77.05	76.02
DotGCN	81.03	78.10	86.16	80.49	76.11	75.00
Semantic-HGCN	78.29	77.16	84.27	77.16	75.78	74.04
APARN	79.91	76.21	87.31	81.79	77.25	75.93
RoBERTa-ACOP	81.57	78.19	86.57	81.11	75.60	74.94
RoBERTa-SDACP	79.78	75.87	85.18	77.66	75.89	75.26
PHNN	81.09	79.23	87.30	81.23	75.49	75.13
RDGCN	80.70	77.54	85.79	80.01	75.92	74.65
TextGT	81.59	78.50	87.31	81.97	**77.26**	**76.18**
**KEMFF**	**83.23**	**80.74**	**88.29**	**83.19**	76.37	76.02

Note: Bold denotes the best result, and underline denotes the second-best result.

**Table 5:** Ablation study results.

Model	Laptop14		Restaurant14		Twitter	
	Acc.	Macro-F1	Acc.	Macro-F1	Acc.	Macro-F1
KEMFF	**83.23**	**80.74**	**88.29**	**83.19**	**76.37**	**76.02**
w/o S	82.91	80.23	87.31	80.81	74.30	72.82
w/o A	82.75	79.36	88.02	82.49	74.45	73.47
w/o K	80.06	76.64	86.77	80.08	74.45	73.77
w/o S + A	79.13	75.28	84.34	76.19	70.22	69.13
w/o S + K	78.27	73.05	84.19	75.33	69.98	68.69
w/o A + K	74.66	72.92	83.81	76.17	69.13	68.34

Note: Bold denotes the best result.

**Table 6:** Fine-grained ablation study of key design choices.

Model	Laptop14		Restaurant14		Twitter	
	Acc.	Macro-F1	Acc.	Macro-F1	Acc.	Macro-F1
KEMFF	**83.23**	**80.74**	**88.29**	**83.19**	**76.37**	**76.02**
w/o at	82.78	79.44	88.07	82.61	75.35	74.57
w/o ff	80.21	78.54	86.13	81.17	74.35	73.89

Note: Bold denotes the best result.

**Table 7:** Mean and standard deviation results over five runs. Statistical significance is tested between KEMFF and the strongest baseline under the same five random seeds.

Model	Laptop14		Restaurant14		Twitter	
	Acc.	Macro-F1	Acc.	Macro-F1	Acc.	Macro-F1
DualGCN	80.08±1.18	77.26±0.64	85.56±0.85	79.51±0.67	74.38±1.41	73.59±1.35
APARN	78.92±0.95	75.32±0.81	86.67±0.59	80.88±0.60	74.82±1.50	73.36±1.51
PHNN	79.98±0.87	77.97±1.12	85.85±0.93	79.31±1.41	73.33±1.51	73.22±1.12
TextGT	80.72±0.65	77.34±0.83	86.26±0.84	80.97±0.82	76.00±0.84	75.05±0.72
KEMFF	82.81±0.54 *	79.62±0.97 *	87.77±0.42 *	82.30±0.83 *	75.35±1.00	74.66±1.33
*p*-value	0.0024	0.0309	0.0191	0.0265	–	–

Note: Bold denotes the best result. * indicates that KEMFF significantly outperforms the strongest baseline under a paired two-tailed Student’s *t*-test with p<0.05.

**Table 8:** Comparison of different external knowledge sources on three datasets.

Knowledge Source	Laptop14		Restaurant14		Twitter	
	Acc.	Macro-F1	Acc.	Macro-F1	Acc.	Macro-F1
Random Knowledge	77.06	75.41	78.49	73.54	63.01	62.55
Shuffled Knowledge	78.47	75.59	83.83	78.80	67.75	64.86
WordNet	83.23	80.74	**88.29**	**83.19**	**76.37**	**76.02**
SentiWordNet	77.96	78.84	87.74	80.43	68.47	65.61
SenticNet	**83.97**	**81.27**	84.97	81.25	66.07	64.96

Note: Bold denotes the best result.

**Table 9:** Case study predictions.

Aspect	Sentence	AOA	BERT_SPC	SSEGCN	Ours	Label
food, service	Great food but service was dreadful!	(N, N)	**(P, N)**	**(P, N)**	**(P, N)**	(P, N)
Windows 8	Biggest complaint is Windows 8.	(O)	**(N)**	**(N)**	**(N)**	(N)
settings	The settings are not user-friendly either.	**(N)**	**(N)**	**(N)**	**(N)**	(N)
i5	Not as fast as I would have expect for an i5.	(P)	(P)	**(N)**	**(N)**	(N)
chicken, taste	In my burrito, here was nothing but dark chicken that had that cooked last week and just warmed up in a microwave taste.	(N, P)	(N, O)	(N, P)	**(N, N)**	(N, N)

**Table 10:** Representative error cases of KEMFF on the Twitter dataset.

Sentence	Aspect	Gold	Pred.	Possible Reason
“can someone explain to me how the hell barack obama won a nobel peace prize, and for what exactly, maybe i’ll win one next year then”	barack obama	Negative	Neutral	The sentence expresses negative sentiment through a rhetorical question and implicit sarcasm, which is difficult for the model to detect from surface-level semantics.
“watching a madonna concert, they are really cool, and she sings awesome in live:)”	madonna	Positive	Neutral	The sentence contains clear positive expressions such as “really cool” and “awesome”, but the informal structure and noisy wording may weaken the sentiment signal.
“obama updates: cutting latin america trip short; nobel peace prize under attack, nobel peace”	obama	Neutral	Negative	News-style text contains negative event words such as “under attack”, which may mislead the model although the sentence is mainly factual.
“last kiss by taylor swift is like the saddest song i’ve ever heard: -lsb-”	taylor swift	Negative	Positive	The model may associate the artist name and song-related context with positive preference, but it fails to capture the negative emotional cue “saddest”.
“watching oprah with goldie hawn regarding happiness should be interesting”	oprah	Positive	Neutral	The positive sentiment is implicit and expressed through a mild phrase “should be interesting”, which is weaker than explicit sentiment words such as “love” or “great”.

## Data Availability

All original contributions of this study are incorporated in this article; any further inquiries may be directed to the corresponding author.

## References

[1] Shukla P., Kumar R., Dwivedi V.K., Singh A.K. (2026). Aspect based sentiment analysis: A systematic review, taxonomy, applications, and future research directions. Comput. Sci. Rev..

[2] Pontiki M., Galanis D., Papageorgiou H., Androutsopoulos I., Manandhar S. SemEval-2016 Task 5: Aspect based sentiment analysis. Proceedings of the 10th International Workshop on Semantic Evaluation (SemEval-2016).

[3] Wang Y., Huang M., Zhu X., Zhao L. Attention-based LSTM for aspect-level sentiment classification. Proceedings of the 2016 Conference on Empirical Methods in Natural Language Processing.

[4] Ma D., Li S., Zhang X., Wang H. Interactive attention networks for aspect-level sentiment classification. Proceedings of the International Joint Conference on Artificial Intelligence (IJCAI).

[5] Zhang C., Li Q., Song D. Aspect-based sentiment classification with aspect-specific graph convolutional networks. Proceedings of the 2019 Conference on Empirical Methods in Natural Language Processing and the 9th International Joint Conference on Natural Language Processing (EMNLP-IJCNLP).

[6] Li R., Chen H., Feng F., Ma Z., Wang X., Hovy E. Dual graph convolutional networks for aspect-based sentiment analysis. Proceedings of the 59th Annual Meeting of the Association for Computational Linguistics and the 11th International Joint Conference on Natural Language Processing (Volume 1: Long Papers).

[7] Zhou J., Huang J.X., Hu Q.V., He L. (2020). SK-GCN: Modeling syntax and knowledge via graph convolutional network for aspect-level sentiment classification. Knowl.-Based Syst..

[8] Liu W., Zhou P., Zhao Z., Wang Z., Ju Q., Deng H., Wang P. K-BERT: Enabling language representation with knowledge graph. Proceedings of the AAAI Conference on Artificial Intelligence.

[9] Devlin J., Chang M.W., Lee K., Toutanova K. BERT: Pre-training of deep bidirectional transformers for language understanding. Proceedings of the 2019 Conference of the North American Chapter of the Association for Computational Linguistics: Human Language Technologies, Volume 1 (Long and Short Papers).

[10] Liu Y., Ott M., Goyal N., Du J., Joshi M., Chen D., Levy O., Lewis M., Zettlemoyer L., Stoyanov V. (2019). RoBERTa: A robustly optimized BERT pretraining approach. arXiv.

[11] Wang Z., Zhang B., Yang R., Guo C., Li M. DAGCN: Distance-based and aspect-oriented graph convolutional network for aspect-based sentiment analysis. Proceedings of the Findings of the Association for Computational Linguistics: NAACL 2024.

[12] Yin S., Zhong G. TextGT: A double-view graph transformer on text for aspect-based sentiment analysis. Proceedings of the AAAI Conference on Artificial Intelligence.

[13] Zhao X., Peng H., Dai Q., Bai X., Peng H., Liu Y., Guo Q., Yu P.S. RDGCN: Reinforced dependency graph convolutional network for aspect-based sentiment analysis. Proceedings of the ACM International Conference on Web Search and Data Mining (WSDM).

[14] Chen H., Zhai Z., Feng F., Li R., Wang X. Enhanced multi-channel graph convolutional network for aspect sentiment triplet extraction. Proceedings of the 60th Annual Meeting of the Association for Computational Linguistics (Volume 1: Long Papers).

[15] Mei X., Zhou Y., Zhu C., Wu M., Li M., Pan S. (2023). A disentangled linguistic graph model for explainable aspect-based sentiment analysis. Knowl.-Based Syst..

[16] Chen Y., Zhang K., Hu F., Wang X., Li R., Liu Q. Dynamic multi-granularity attribution network for aspect-based sentiment analysis. Proceedings of the 2024 Conference on Empirical Methods in Natural Language Processing.

[17] Ma F., Hu X., Liu A., Yang Y., Li S., Yu P.S., Wen L. AMR-based network for aspect-based sentiment analysis. Proceedings of the 61st Annual Meeting of the Association for Computational Linguistics (Volume 1: Long Papers).

[18] Jin Z., Tao M., Wu X., Zhang H. (2024). Span-based dependency-enhanced graph convolutional network for aspect sentiment triplet extraction. Neurocomputing.

[19] Hossain M.M., Sanjara, Hossain M.S., Chaki S., Rahman M.S., Shawkat Ali B.M.S. (2025). Enhancing sentiment analysis with local and global memory in heterogeneous graph neural networks. Knowl.-Based Syst..

[20] Cheng Y., Yuan M., He F., Shao X., Wu J. (2025). Aspect-based sentiment analysis based on multi-granularity graph convolutional network. Neural Netw..

[21] Cambria E., Speer R., Havasi C., Hussain A. SenticNet: A publicly available semantic resource for opinion mining. Proceedings of the AAAI Fall Symposium on Commonsense Knowledge.

[22] Miller G.A. (1995). WordNet: A lexical database for english. Commun. ACM.

[23] Baccianella S., Esuli A., Sebastiani F. SentiWordNet 3.0: An enhanced lexical resource for sentiment analysis and opinion mining. Proceedings of the Seventh International Conference on Language Resources and Evaluation (LREC’10).

[24] Zhong Q., Ding L., Liu J., Du B., Jin H., Tao D. (2023). Knowledge graph augmented network towards multiview representation learning for aspect-based sentiment analysis. IEEE Trans. Knowl. Data Eng..

[25] Wan B., Wu P., Han P., Li G. (2025). Aspect-based sentiment analysis by knowledge and attention integrated graph convolutional network. Appl. Soft Comput..

[26] Liu H., Wu Y., Li Q., Lu W., Li X., Wei J., Liu X., Feng J. (2023). Enhancing aspect-based sentiment analysis using a dual-gated graph convolutional network via contextual affective knowledge. Neurocomputing.

[27] Liu H., Li X., Lu W., Cheng K., Liu X. (2024). Graph augmentation networks based on dynamic sentiment knowledge and static external knowledge graphs for aspect-based sentiment analysis. Expert Syst. Appl..

[28] Ji J., Zhu W., Hou C., Song Q., Ma Y., Wang Y. (2026). DAGF: A dual GCN and auxiliary graph fusion based model for aspect-based sentiment analysis. Appl. Soft Comput..

[29] Shi X., Ding W., Hu M., Kang X., Ren F. (2025). Triple dimensional psychology knowledge encouraging graph attention networks to exploit aspect-based sentiment analysis. Sci. Rep..

[30] Zhong X., Di H., Zhao X., Bai C., Zhong Z. (2026). Multi-granularity features fusion with hierarchical networks for aspect-based sentiment analysis. Electron. Res. Arch..

[31] Mrini K., Dernoncourt F., Tran Q.H., Bui T., Chang W., Nakashole N. Rethinking self-attention: Towards interpretability in neural parsing. Proceedings of the Findings of the Association for Computational Linguistics: EMNLP 2020.

[32] Bevilacqua M., Blloshmi R., Navigli R. One SPRING to rule them both: Symmetric AMR semantic parsing and generation without a complex pipeline. Proceedings of the AAAI Conference on Artificial Intelligence.

[33] Han X., Cao S., Lv X., Lin Y., Liu Z., Sun M., Li J. OpenKE: An open toolkit for knowledge embedding. Proceedings of the 2018 Conference on Empirical Methods in Natural Language Processing: System Demonstrations.

[34] Dong L., Wei F., Tan C., Tang D., Zhou M., Xu K. Adaptive recursive neural network for target-dependent Twitter sentiment classification. Proceedings of the 52nd Annual Meeting of the Association for Computational Linguistics (Volume 2: Short Papers).

[35] Huang B., Ou Y., Carley K.M. Aspect level sentiment classification with attention-over-attention neural networks. Proceedings of the Social, Cultural, and Behavioral Modeling.

[36] Song Y., Wang J., Jiang T., Liu Z., Rao Y. (2019). Attentional encoder network for targeted sentiment classification. arXiv.

[37] Zeng B., Yang H., Xu R., Zhou W., Han X. (2019). LCF: A local context focus mechanism for aspect-based sentiment classification. Appl. Sci..

[38] Zhang Z., Zhou Z., Wang Y. SSEGCN: Syntactic and semantic enhanced graph convolutional network for aspect-based sentiment analysis. Proceedings of the 2022 Conference of the North American Chapter of the Association for Computational Linguistics: Human Language Technologies.

[39] Chen C., Teng Z., Wang Z., Zhang Y. Discrete opinion tree induction for aspect-based sentiment analysis. Proceedings of the 60th Annual Meeting of the Association for Computational Linguistics (Volume 1: Long Papers).

[40] Wu Z., Zhao H., Gu T., Cao G. Semantic task-assisted aspect-based sentiment analysis. Proceedings of the 22nd Chinese National Conference on Computational Linguistics.

[41] Zeng Y., Li Z., Chen Z., Ma H. (2023). Aspect-level sentiment analysis based on semantic heterogeneous graph convolutional network. Front. Comput. Sci..

[42] Zhu W., Luo J., Miao Y., Liu P. (2023). PHNN: A prompt and hybrid neural network-based model for aspect-based sentiment classification. Electronics.

